# Investor memory of past performance is positively biased and predicts overconfidence

**DOI:** 10.1073/pnas.2026680118

**Published:** 2021-09-02

**Authors:** Daniel J. Walters, Philip M. Fernbach

**Affiliations:** ^a^INSEAD, Singapore 138676;; ^b^Leeds School of Business, University of Colorado Boulder, Boulder, CO 80309

**Keywords:** overconfidence, investor behavior, memory bias, trading frequency

## Abstract

We document a memory-based mechanism associated with investor overconfidence. In Studies 1 and 2, investors were asked to recall their most important trades in the recent past and then reported investing confidence and trading frequency. After the study, they looked up and reported the actual returns of these trades. In both studies, investors were biased to recall returns as higher than achieved, and larger memory biases were associated with greater overconfidence and trading frequency. The design of Study 2 allowed us to separately investigate the effects of two types of memory biases: distortion and selective forgetting. Both types of bias were present and were independently associated with overconfidence and trading frequency. Study 3 was an incentive-compatible experiment in which overconfidence and trading frequency were reduced when participants looked up previous consequential trades compared to when they reported them from memory.

## Significance

This paper makes several contributions to research in memory, overconfidence, and investment behavior. First, we find that investors’ memories for past performance are positively biased. They tend to recall returns as better than achieved and are more likely to recall winners than losers. No published paper has shown these effects with investors. Second, we find that these positive memory biases are associated with overconfidence and trading frequency. Third, we validated a new methodology for reducing overconfidence and trading frequency by exposing investors to their past returns.

Overconfidence is hazardous to your wealth. Overconfident investors trade frequently despite losing money doing so (1), take on too much leverage (2), overreact to market signals (3, 4), and suffer from the “winner’s curse” in which they purchase overpriced investments (5). Overconfident investors are also more likely to commit investment errors such as under-diversification and overconcentration on familiar stocks (6).

One possible explanation for investor overconfidence is that investors are biased to recall their trading performance as better than actually achieved. This possibility has been suspected by practitioners for some time. For instance, Erik Davidson, the chief investment officer for Wells Fargo, provided his intuition for the source of investor overconfidence: “Much like our human predisposition toward nostalgia about the past, where we only remember the good times and gloss over the bad, investors likewise tend to take a nostalgic view of their past winners but forget about their past losing investments” (7). Similarly, theoretical economic models have suggested that positively biased memory could contribute to overconfidence (8), and laboratory evidence suggests participants have stronger memories for positive versus negative financial outcomes (9). More generally, research has shown that people fill in gaps in memory using unreliable cues, which can lead to overconfidence (10, 11).

Overconfidence is usually explained by appealing to information processing biases such as confirmation bias (12–14), positive test strategy (15–17), wishful thinking (18), motivated reasoning (19), and failure to consider unknowns (20). We propose that overconfidence can also be driven by positivity biases in memory for past performance. In the domain of investing, positivity biases could take two forms: distortion and selective forgetting. Distortion means that the magnitude of returns is remembered as better than reality; winners are remembered more positively and losers less negatively. Selective forgetting means that consequential losing trades are less likely to be recalled than consequential winning trades.

Distortion and selective forgetting have both been documented outside of investing but not linked to overconfidence (21–23). For instance, distortion was found among college students who remembered their high school grades as being higher than they achieved in reality (24, 25) and among patients who recalled their cholesterol scores and cardiovascular risk categories as more favorable than shown on a recently viewed test (26).

Selective forgetting was found in study participants who tended to more readily forget details about negative feedback than equivalent positive feedback on a performance evaluation (27). Researchers have suggested this bias occurs because people reminisce more on positive information than on negative information following ego-enhancement motivations (28, 29). Selective forgetting becomes stronger with age (30) but also occurs in children as young as 5 y old (31).

In all studies, we recruited real investors and had them report consequential trades, their confidence level in terms of their perceived ability to beat the market, and their trading frequency or intention to trade frequently. We examine trading frequency because it is both widely viewed as costly to investors and because it is consistently linked to investor overconfidence (1, 6, 32). Study 1 demonstrates a positive memory bias among real investors and shows that investors with larger memory biases are more overconfident and trade more frequently in a correlational design. In Study 2, we replicate these effects in a more elaborate design that allows us to distinguish effects of distortion from selective forgetting. In Study 3, we demonstrate a causal effect of memory bias on overconfidence and on an incentive-compatible measure of intention to trade frequently. Participants reported consequential trades either from memory or by looking them up. Overconfidence and trading intentions were mitigated in the latter case.

All three experiments were approved by the European Institute of Business Administration (INSEAD) Institutional Review Board (IRB) at the INSEAD Sorbonne Université Behavioral Laboratory (https://www.insead.edu/centres/insead-sorbonne-universite-lab-en). The participants gave informed consent at the start of each experiment after reading an IRB-approved consent form. Methods, predictions, and analyses for all studies were determined prior to data collection and preregistered on https://aspredicted.org. IRB documentation and study preregistrations can be found in *SI Appendix*.

## Study 1

We recruited real investors through Pollfish, an online polling company. Participants were required to be active investors that have $1,000 or more in stock market investments, have two or more individual stocks held in 2019, and have access to their trading history in 2019. Participants recalled their two most consequential investments over the past year from memory, examined their financial statement to report the actual returns of the two most consequential investments, and completed a measure of overconfidence (32) and trading frequency (6). We predicted that investors would show a positivity bias such that the average recalled returns would be greater than their objective returns. We also predicted that a positivity bias would be associated with overconfidence and trading frequency.

## Methods

We recruited 411 participants in the United States (33.5% female; *M*_age_ = 37.0; SD = 12.7 y) for $2.00 each on Pollfish. Descriptive statistics are included in Table 1. Participants completed the following three tasks in a randomized order.

**Table 1.** Descriptive statistics for Studies 1, 2, and 3

	Study 1	Study 2	Study 3
Observations	411	151	366
Overconfidence			
Mean (SD)	13.0% (24.3%)	8.8% (27.9%)	7.6% (11.0%)
Median	5.0%	2.7%	5.0%
Trading frequency*			
Median	1 trade per month	1 trade per week	10 trades per month
75th percentile	1 trade per week	2 trades per week	20 trades per month
25th percentile	1 trade per quarter	2 trades per month	5 trades per month
Income^†^			
Median	$75,000 to $99,999	$50,000 to $74,999	$50,000 to $74,999
75th percentile	$100,000 to $149,999	$100,000 to $149,999	$75,000 to $99,999
25th percentile	$50,000 to $74,999	$25,000 to $49,999	$25,000 to $49,999
Education^‡^			
Median	Bachelor’s degree	Bachelor’s degree	Bachelor’s degree
75th percentile	Bachelor’s degree	Master’s degree or higher	Master’s degree or higher
25th percentile	Associate degree	Associate degree	Associate degree
Stocks owned (open response)			
Median	5	15	7
75th percentile	15	29	20
25th percentile	3	7	3
Asset value^¶^			
Median	$5,000 to $9,9999	$1,000 to $4,999	$5,000 to $9,9999
75th percentile	$25,000 to $49,000	$10,000 to $29,999	$25,000 to $49,000
25th percentile	$500 to $999	$100 to $499	$1,000 to $4,999

* Trading frequency measurement scale differed in each study and is provided in *Methods*.

^†^ Income measured on a seven-point scale (1: $0 to $24,999, 2: $25,000 to $49,999, 3: $50,000 to $74,999, 4: $75,000 to $99,999, 5: $100,000 to $149,999, 6: $150,000 to $199,999, and 7: $200,000 or more).

^‡^ Education measured on a five-point scale (1: less than high school, 2: high school, 3: associate degree, 4: bachelor’s degree, and 5: master’s degree or higher).

^¶^ Asset value measured on a 10-point scale (1: $0 to $99, 2: $100 to $499, 3: $500 to $999, 4: $1,000 to $4,999, 5: $5,000 to $9,999, 6: $10,000 to $24,999, 7: $25,000 to $49,999, 8: $50,000 to $99,999, 9: $100,000 to $499,999, and 10: $500,000 or more).

### Memory Phase: Recalled Investment Returns.

Participants were instructed to recall the stock investments that had the largest monetary impact (gains or losses) on their portfolio:

“Please recall the two stock investments that have had the biggest monetary impact on your investment portfolio in 2019 (i.e., since January 1, 2019). These can be stocks where you lost money or gained money. What's important is that these are the two stock investments that had the biggest monetary impact on your portfolio.”

“Please write down the two stocks in the order of monetary impact. Stock 1 should have the largest impact, and Stock 2 should have the second largest impact. For instance, if an investment in Apple stock had the largest monetary impact and Walmart stock had the second largest, then you would write down your year-to-date return in Apple for ‘Stock 1’ and in Walmart for ‘Stock 2.’”

Participants were instructed not to reference any outside material and to only use their memories. Participants provided the name and percentage return for both investments in free-response boxes. Inputs could take values between −250 and 250%.

### Overconfidence.

To assess overconfidence, we used an established measure of investor overplacement in which participants estimated their ability to outperform the market over the following 12 mo (6, 32). Overplacement, also known as the better-than-average effect, occurs when people overestimate their performance relative to others. Overplacement is viewed as one of the three primary forms of overconfidence alongside overprecision, which is overconfidence about estimate precision, and overestimation, which is overconfidence about absolute performance (33). Relative market performance was validated as a measure of overplacement based on the assumption that the overall market return sets the average an investor can expect to earn and any expected outperformance means to be better than this average (6). The measure was further verified to be highly correlated with a second measures of overplacement that takes portfolio risk into account* and a measure of overestimation in which an investor’s actual market return was subtracted from their estimates of their own market return over this same period (32). In the present study, investors were asked the following:

“Consider the next 12 mo of trading and estimate by how many percentage points you will outperform or underperform the general market return. For instance, if you expect to outperform the market by 10%, then you would respond 10%. If you expect to underperform the S&P 500 by 10%, then you would respond −10%. Please respond below.”^†^

### Trading Frequency.

Participants were asked a previously established trading frequency measure (6)^‡^: “How often do you trade in the financial markets” (1: at least once a day, 2: at least once a week, 3: at least once a month, 4: at least once a quarter, 5: at least once a year, and 6: less than once a year). This measure was reverse coded so that higher ratings were associated with more frequent trading. Participants recalled returns, assessed overconfidence, and rated trading frequency on three separate pages in an order that was randomized for each participant. We detected no differences by order.

### Statement Phase: Actual Investment Returns.

Participants were then asked to provide their objective investment returns from their financial statements: “Please answer the following question again, except this time, we want you to open up your trading statement to answer as accurately as possible.” Participants were also shown the instructions from the memory phase and provided the returns for the two stocks that had the biggest impact on their portfolios in free-response boxes.

To encourage truthful responding, participants were told they could receive a bonus for accurate reporting. Participants were first asked how much they would need to be paid to submit anonymized versions of their financial statements (up to $200). Participants were told that we would be randomly selecting some participants to have an anonymized version of their financial statement reviewed, but the participant would only be paid if the information reported was correct to a single percentage point. This statement was on the same page that participants reported their returns.

Participants were then asked if they reported the same stocks in part one and part two. Participants then provided control and demographic variables including age, gender, income, total investment assets, portfolio composition, number of stocks owned, and whether they have a financial advisor. As a truthfulness check, we asked participants if they accessed their financial statements in the previous section. Participants were then debriefed.

## Results

A total of 88.6% of participants reported that they were able to access their financial statement, and 73.0% of participants agreed to share their statements for verification. The following results do not substantively change if the participants that were unable to access their financial statements are excluded from analysis. The average price at which these participants were willing to share was $79.73. After the study, we paid two participants to show us their financial statements and verified that they did indeed accurately report their returns.

### Memory Bias.

We tested for memory bias by comparing the actual return and the return reported from memory of the two investments separately. For both investments, there was a positive memory bias such that the investment from memory yielded a higher return on average [Trade 1: M_memory_ = 44.1%, SD = 64.5%; M_actual_ = 39.8%, SD = 60.0%, *t*(410) = 2.14, *P* = 0.033, *d* = 0.15; Trade 2: M_memory_ = 40.6%, SD = 61.8%; M_actual_ = 33.5%, SD = 56.0%, *t*(410) = 3.43, *P* < 0.001, *d* = 0.24]. Additional robustness checks are in *SI Appendix*.

### Overconfidence.

Investors in this sample predicted that they would outperform the S&P 500 by 13.0% on average (SD = 24.3%). This was greater than 0%, *t*(410) = 10.81, *P* < 0.001, *d* = 0.77, indicating overconfidence. We next examined the primary prediction that investors with a positivity bias in recalled returns would be more overconfident. We tested this in a robust regression with positivity bias as the independent variable and overconfidence as the dependent variable while clustering SEs by participant. Positivity bias was calculated at the individual level as the average return reported in the statement phase subtracted from the average return reported in the memory phase. As predicted, the degree of positivity bias was associated with greater overconfidence (Table 2, column 1). To give an intuition for the effect size, Cohen’s *d* was equal to 0.71, and the predicted overconfidence 1 SD above the mean positivity bias was 21.1% compared to 13.0% at the mean. This pattern of results held when adding control variables of age, gender, income, total investment assets, number of stocks owned, and whether they have a financial advisor (Table 2, column 2).

**Table 2.** Study 1 positivity bias predicts overconfidence and trading frequency

Dependent variable	Overconfidence		Trading frequency	
	(1)	(2)	(3)	(4)
Positivity bias	0.197**	0.200***	0.352*	0.356**
	(0.062)	(0.057)	(0.137)	(0.132)
Gender (1: male, 2: female)		0.004		−0.392**
		(0.022)		(0.144)
Education		−0.017		−0.039
		(0.015)		(0.077)
Age		−0.000		−0.026***
		(0.001)		(0.006)
Financial advisor (1: yes, 2: no)		−0.030		−0.468**
		(0.021)		(0.145)
Asset value		−0.002		−0.018
		(0.006)		(0.045)
Income		0.007		0.175**
		(0.010)		(0.065)
Number of stocks owned		0.000		0.000***
		(0.000)		(0.000)
Constant	11.847***	20.226**	4.116***	5.745***
	(1.013)	(7.622)	(0.073)	(0.467)
				
Observations	822	776	822	776

Robust SEs are in parentheses. All estimates represent ordinary least squares regression coefficients. Overconfidence and positivity bias coded on a decimal basis (e.g., 10% coded as 0.1). +*P* < 0.01, *P < 0.05, **P < 0.01, and ***P < 0.001.

### Trading Frequency.

We then examined the prediction that investors with a larger positivity bias in recalled returns would trade more frequently. We tested this in a robust regression with bias as the independent variable and trading frequency as the dependent variable while clustering SEs by participant. Confirming this prediction, the degree of positivity bias was associated with greater trading frequency (Table 2, column 3). To give an intuition for the effect size, Cohen’s *d* was equal to 0.20. This pattern of results held when adding control variables of age, gender, income, total investment assets, number of stocks owned, and whether they have a financial advisor (Table 2, column 4).

### Mediation Analysis.

We next conducted an exploratory analysis^§^ to examine whether the relationship between positivity bias and trading frequency could be statistically explained by overconfidence. We tested this using a structural equation model with trading frequency as the dependent variable, positivity bias as the independent variable, and overconfidence as the mediator variable. We calculated indirect pathways using bootstrapped SEs (10,000 resamples). We found a statistically reliable indirect effect through overconfidence, b = 0.268, bias-corrected 95% CI = [0.090, 0.503], *P* = 0.011. Refer to *SI Appendix* for additional details and assumptions.

## Discussion

In this study, investors recalled past returns as higher than achieved, and this bias predicted both overconfidence and more frequent trading. In a mediation analysis, overconfidence statistically mediated the relationship between memory bias and trading frequency.

## Study 2

One limitation of Study 1 is that participants only reported two investments, and we could therefore only do a coarse analysis of memory bias that does not distinguish distortion from selective forgetting. Moreover, by asking participants to recall a larger number of investments, we can obtain a more ecologically valid estimate of the effect size of memory bias. In Study 2, experienced investors first recalled the 10 trades that had the largest monetary impact on their portfolio in 2020. They then completed measures of overconfidence and trading frequency. Finally, they reported the 10 trades in 2020 that had the biggest impact on their portfolio from their financial statements. With a larger sample of trades from each participant, we were able to separately calculate measures of distortion and selective forgetting and test whether they predict overconfidence and trading frequency.

## Methods

We recruited 3,167 participants on Prolific Academic who were then screened for having $1,000 or more in stock market investments, having made 10 or more trades in 2020, and having access to their financial statements. This yielded 501 qualified participants that were offered the opportunity to participate in our study. From this pool of qualified candidates, we recruited 151 participants for £4.00 each. We closed the survey after 3 d when we reached our preregistered sample size of 150. Seven participants did not complete the survey, leaving us with 144 participants (26.4% female; *M*_age_ = 36.2; SD = 12.2 y). Descriptive statistics are included in Table 1.

### Memory Phase: Recalled Investment Returns.

Participants were instructed to recall the 10 trades that had the largest monetary impact (gains or losses) on their portfolio in the first 6 mo of 2020:

“Complete the next task using only your memory. Please don't look anything up. It may be difficult to remember everything. That's fine. Just do your best. Please think back on the trades you made in 2020 that had the biggest impact on your portfolio. These should be trades that you bought after January 1, 2020 and sold before July 1, 2020. These can be trades where you made or lost money. These can be trades in any stock asset, mutual fund, or stock derivative.”^¶^

Participants could enter up to 10 trades but could leave trades blank if they could not remember. Participants entered 7.0 trades on average (SD = 3.3). After entering the trades, participants provided the dollar value of the purchase and sale price of each trade, each on a separate page. We modified this elicitation from Study 1 in which participants reported a percentage return to increase the methodological generalizability of the research.

### Overconfidence.

To assess overconfidence, participants estimated their ability to outperform the market over the following 12 mo. In Study 1, participants provided a single estimate of market outperformance. In the present study, participants first estimated the return of the S&P 500 over the following 12 mo. They then estimated the investment return of their own portfolio. Overconfidence was calculated as their own performance subtracted from their forecast of the S&P 500. This was changed to more closely follow the methodology of Merkle (2017) who also measured overconfidence by subtracting investors’ expected performance from their market forecast.

### Trading Frequency.

Participants were asked a trading frequency question: “How often do you trade in the financial markets?” This measure was similar to Study 1, except that it had 10 scale points versus six points, allowing for a greater resolution. Refer to *SI Appendix* for a complete scale. This measure was coded so that higher ratings were associated with more frequent trading.

### Statement Phase: Actual Investment Returns.

Participants were then asked to identify their actual top 10 trades from their financial statements as in Study 1. Participants were asked the following:

“Please open your financial statement for 2020. Please use only your financial statements to complete this task. Please examine your financial statement and identify the trades you made in 2020 that had the biggest impact on your portfolio. These should be trades that you bought after January 1, 2020 and sold before July 1, 2020. These can be trades where you made or lost money.” Participants entered 8.2 trades on average (SD = 2.8).

Participants then matched each of the trades listed by memory to the trades listed by statement in a dropdown menu. Participants also had the option to say that a trade they had listed from memory was not on the list when working off the statements (i.e., when a trade was mistakenly remembered as being in the top 10).

Participants then provided the percent return for each of the trades listed from their statements. Participants also provided the dollar value of each investment, the investment start date, and the investment end date. Participants were instructed to record this information directly from their financial statements. Participants also provided the same information from the financial statements for trades that had been identified in the memory phase but did not appear on the top 10 in the statement phase.

As a truthfulness check, participants were then asked if they had used their financial statements to complete part two. Participants then provided their age, gender, income, education, total investment assets, number of stocks owned, and whether or not they have a financial advisor and were then debriefed.

## Results

A total of 95.8% of participants reported that they did use their financial statements to complete part two. The following results do not substantively change if the participants that reported not using their financial statements are excluded from the analysis.

### Overall Positivity Bias.

We first examined whether investors showed an overall bias to recall higher returns in the memory phase compared to statement phase using an analogous analysis to S1. For each participant, we first averaged the return of the top 10 stocks reported in the memory phase and then averaged the return of the top 10 stocks reported in the statement phase. Participants reported a higher average return in the memory phase (M = 29.6%, SD = 28.2) than in the statement phase [M = 21.6% SD = 41.2%, *t*(143) = 3.54, *P* < 0.001, *d* = 0.30]. We performed a more comprehensive statistical test by running a mixed linear regression with the trade return as the dependent variable and whether the trade was reported from memory or from statement as the independent variable, with SEs clustered at the participant level and at the individual stock level. Trades had higher returns when reported from memory (Table 3, column 1). This pattern of results held when adding control variables of age, gender, income, total investment assets, number of stocks owned, and whether they have a financial advisor (Table 3, column 2).

**Table 3.** Study 2 distortion and selective forgetting

Dependent variable	Trade return				Trade forgotten	
					0: remembered, 1: forgotten	
	(1)	(2)	(3)	(4)	(5)	(6)
Condition (0: memory, 1: statement)	−0.088***	−0.086***	−0.037*	−0.037*		
	(0.018)	(0.018)	(0.016)	(0.017)		
Return valence (0 = negative, 1 = positive)					−0.435*	−0.435*
					(0.186)	(0.185)
Absolute percentage return						−0.586**
						−0.000**
Dollar value of trade		0.000		0.000		(0.000)
		(0.000)		(0.000)		−0.001
Number of days the position was held		0.001		0.001+		(0.001)
		(0.000)		(0.000)		−0.000
Number of days since trade closed		0.000		0.000		(0.001)
		(0.000)		(0.000)		0.299
Gender (1: male, 2: female)		−0.186***		−0.201***		(0.287)
		(0.052)		(0.057)		−0.014
Age		−0.006**		−0.007**		(0.011)
		(0.002)		(0.002)		−0.114
Income		−0.006		−0.006		(0.081)
		(0.024)		(0.026)		−0.018
Education		0.031		0.026		(0.137)
		(0.036)		(0.040)		0.146*
Asset value		−0.009		−0.009		(0.070)
		(0.022)		(0.023)		−0.000
Number of stocks owned		0.000		0.000		(0.000)
		(0.000)		(0.000)		0.620+
Financial advisor (1: yes, 2: no)		0.004		0.028		(0.369)
		(0.067)		(0.070)		(0.219)
Constant	0.394***	0.651***	0.345***	0.605**	−0.417*	−1.223
	(0.046)	(0.193)	(0.041)	(0.216)	(0.168)	(1.065)
Observations	2,397	2,394	2,010	2,010	1,161	1,161

Robust SEs are in parentheses. Estimates in columns 1 to 4 represent ordinary least squares regression coefficients. Estimates in columns 5 and 6 represent log odds coefficients from a logistic regression. SEs are clustered at the trade and participant level in columns 1 to 4 and at the participant level in columns 5 and 6). +*P* < 0.01, *P < 0.05, **P < 0.01, and ***P < 0.001.

### Distortion.

We then examined whether there was evidence of memory bias from distortion (i.e., recalling the return of a particular trade as better than actually achieved). Our design allowed us to separate out the effect of distortion by observing how the return reported for the same trade changed between the memory phase into the statement phase. For instance, a participant may have reported a trade in Google stock on May 1 in the memory phase and then report that same trade in the statement phase. If the return reported in the memory phase was higher than the statement phase, this would be evidence in support of a distortion bias.

As a first test of distortion, we calculated the difference between the return reported in the memory phase and the return reported in the statement phase for all stocks reported in both phases. We matched the trades identified in the memory phase to the trades in the statement phase using participant’s self-reports of whether they were the same trade. The average distortion at the participant level (M = 4.4%, SD = 20.3%) was significantly greater than 0%, *t*(143) = 2.58, *P* = 0.011.

We next performed a more comprehensive statistical test by running a repeated measures regression with the trade return as the dependent variable and whether the trade was reported from memory or from statement (0: memory, 1: statement) as the independent variable, with SEs clustered at the participant level and at the individual stock level. Participants recalled a trade as having a higher return when the return was reported in the memory phase (M = 30.6%, SD = 65.3%) than when the same trade return was reported in the statement phase (M = 27.0%, SD = 68.7%; Table 3, column 3). This pattern of results held when adding control variables of the dollar value of the trade, the number of days the position was held, the number of days since the trade was closed, gender, age, income, education, total investment assets, number of stocks owned, and whether the participant has a financial advisor (Table 3, column 4).

### Selective Forgetting.

We next examined whether there was evidence of memory bias from selective forgetting (i.e., being more likely to forget losses than gains). To do this, we examined whether the trades reported in the statement phase were remembered or forgotten during the memory phase and whether the associated return reported in the statement phase was a gain or a loss. Our sample consisted of 1,161 trades listed in the statement phase. Within this sample of trades, 784 (67.5%) were remembered, 377 (32.4%) were forgotten, 859 (74.0%) were gains, and 302 (26.0%) were losses.^#^

As a first test of selective forgetting, we calculated the proportion of loss trades to gain trades in the memory and statement phases by participant.^‖^ In the memory phase, participants reported 19.3% losing trades, whereas participants in the statement phase reported 23.6% losing trades, and this difference was significantly greater than 0 (M = 4.3%, SD = 12.9%, z = 4.04, *P* < 0.001). This participant-level measure of selective forgetting was not significantly correlated with the participant-level measure of distortion (i.e., the simple analysis of distortion described above: the average return reported in the memory phase and the return reported in the statement phase for all stocks reported in both phases; *r* = 0.13, *P* = 0.123).

We next performed a more comprehensive statistical test by running a logistic regression with whether the trade was forgotten (0: remembered, 1: forgotten) as the dependent variable and whether the trade was a gain or a loss (0: loss, 1: gain) as the independent variable, with SEs clustered at the participant level. This analysis confirmed that participants forgot their losses at a higher rate (M = 39.7%) than their gains (M = 29.9%; Table 3, column 5). The effect of gain versus loss on being remembered remained significant when controlling for the absolute percentage return of the trade, the dollar value of the trade, the number of days the position was held, the number of days since the trade was closed, gender, age, income, education, total investment assets, number of stocks owned, and whether the participant has a financial advisor (Table 3, column 6).

### Overconfidence.

Participants predicted that the S&P 500 would return 18.1% over the next 12 mo and that they would earn returns of 27.0% over this same time period. Participants’ expected outperformance (M = 8.8%, SD = 27.9%) was significantly greater than 0%, *t*(143) = 3.80, *P* < 0.001, *d* = 0.32, indicating overconfidence. We next examined our prediction that overconfidence would be positively associated with distortion and selective forgetting of trade returns. We tested this in a robust linear regression with overconfidence (i.e., expected outperformance) as the dependent variable and distortion (i.e., the simple analysis of distortion: the difference between the average return reported in the memory phase and the return reported in the statement phase for all stocks reported in both phases) and selective forgetting (i.e., the simple analysis of selective forgetting: the difference in the proportion of loss trades to gain trades in the memory and statement phases)** as independent variables. Distortion and selective forgetting were both significant predictors of overconfidence (Table 4, column 1). To give an intuition for the effect size, Cohen’s *d* was equal to 0.67 for distortion and 0.39 for selective forgetting. Predicted overconfidence 1 SD above the mean distortion level was 17.6% compared to 8.8% at the mean. Predicted overconfidence 1 SD above the mean selective forgetting level was 13.9% compared to 8.8% at the mean. This pattern of results held when adding control variables of gender, age, income, education, total investment assets, number of stocks owned, and whether the participant has a financial advisor (Table 4, column 2).

**Table 4.** Study 2 distortion and selective forgetting predict overconfidence and trading frequency

Dependent variable	Overconfidence		Trading frequency	
	(1)	(2)	(3)	(4)
Distortion	0.434**	0.440*	1.036+	1.513***
	(0.164)	(0.175)	(0.527)	(0.434)
Selective forgetting	0.398**	0.456**	3.150**	3.320**
	(0.132)	(0.141)	(0.972)	(1.080)
Gender (1: male, 2: female)		−0.082*		−1.416***
		(0.035)		(0.316)
Age		−0.000		−0.029+
		(0.002)		(0.015)
Income		0.023		−0.001
		(0.021)		(0.125)
Education		−0.013		−0.050
		(0.028)		(0.184)
Asset value		−0.004		−0.068
		(0.011)		(0.081)
Number of stocks owned		0.000***		0.001*
		(0.000)		(0.000)
Financial advisor (1: yes, 2: no)		−0.096+		−0.024
		(0.050)		(0.397)
Constant	0.052*	0.291*	5.855***	8.884***
	(0.020)	(0.129)	(0.149)	(1.219)
Observations	144	144	144	144

Robust SEs are in parentheses. All estimates represent ordinary least squares regression coefficients. +*P* < 0.01, *P < 0.05, **P < 0.01, and ***P < 0.001.

### Trading Frequency.

We next examined our prediction that trading frequency would be associated with distortion and selective forgetting of trade returns. We tested this in a robust linear regression with trading frequency as the dependent variable and individual-level distortion and individual-level selective forgetting as independent variables. Distortion was marginally significant, and selective forgetting was a significant predictor of trading frequency (Table 4, column 3). To give an intuition for the effect size, Cohen’s *d* was equal to 0.24 for distortion and 0.46 for selective forgetting. Both distortion and selective forgetting were significant predictors when adding control variables of gender, age, income, education, total investment assets, number of stocks owned, and whether the participant has a financial advisor (Table 4, column 4).

### Mediation Analysis.

We next conducted an exploratory analysis to examine whether the relationship between the two types of positivity bias and trading frequency could be statistically explained by overconfidence. We tested this using a two-path structural equation model with trading frequency as the dependent variable for both paths, distortion as the first independent variable, selective forgetting as the second independent variable, and overconfidence as the mediator variable for both paths. We calculated indirect pathways simultaneously using bootstrapped SEs (10,000 resamples). We found a marginally significant indirect effect from distortion through overconfidence, b = 0.76, bias-corrected 95% CI = [−0.02; 1.66], *P* = 0.085, and a significant indirect effect from selective forgetting through overconfidence, b = 0.70, bias-corrected 95% CI = [0.12; 1.45], *P* = 0.042. Refer to *SI Appendix* for additional details and assumptions.

## Discussion

In this study, we found that both distortion and selective forgetting of losses independently predicted overconfidence and trading frequency. These results are consistent with the interpretation that distortion and selective forgetting both influence overconfidence. If it were the case that overconfidence influenced positivity biases (i.e., the reverse casual path) or that a third variable is related to both positivity biases and overconfidence, then we should expect selective forgetting and distortion to share variance in these regressions and to be positively correlated.

## Study 3

The goal of Study 3 was to provide experimental evidence that memory biases lead to overconfidence and increased trading frequency. Experienced investors were recruited to participate in an experiment through online investment forums. The control condition was similar to Studies 1 and 2. In the treatment condition, participants were asked to open their financial statement and report objective stock returns prior to completing the other measures. We predicted that seeing the objective returns, and therefore eliminating memory bias, would mitigate overconfidence and an incentive-compatible measure of trading frequency.

## Methods

We recruited 366 participants (26.4% female; *M*_age_ = 36.2; SD = 12.2 y) through online investment forums. We initially targeted 10 of the largest online investing forums (Analyst forum, City Data, Discuss Personal Finance, HYIP, Morningstar, Quantnet Forum, Reddit (investment section), GuruFocus, Online Traders Forum, and Stock Rants) and were able to successfully post an advertisement on the first six. The post asked for investors who met the following criteria to participate in a “screening study”: 1) at least $1,000 in stock market investments, 2) have owned at least two individual stocks in 2018, and 3) have access to their trading history in 2018. Descriptive statistics for the final sample are included in Table 1. Participants were informed that some of them would be selected to participate in a “larger study” in which they would be given $500 to invest over a 3-mo period (and would be able to keep the value of the investments at the end of this period). This larger study was primarily used as an incentive for truthful participation in the current study. After the experiment, two participants were selected to participate in the larger study and endowed with $500. Upon entering the “screening study” (i.e., the current study), participants were randomly assigned to either a treatment or control condition.

### Treatment Condition.

In the treatment condition, participants were instructed to access their financial statements and write down the two stock investments that had the largest monetary impact (gains or losses) on their portfolio. Following the prompt below, participants wrote down the two stocks and the two associated returns in text boxes:

“Look up the two stock investments that had the largest monetary impact on your investment portfolio in 2018 (i.e., between January 1, 2018 and December 31, 2018). These can be investments where you lost money or gained money. Please only consider investments in individual stocks rather than mutual funds or index funds.”

### Control Condition.

In the control condition, participants were given similar instructions to write down the sectors in which they were invested in 2018 in text boxes:

“Look up the two sectors where you held the largest percentage of your stock investments in 2018 (i.e., between January 1, 2018 and December 31, 2018). These can be sectors where you lost money or gained money. Please only consider investments in individual stocks rather than mutual funds or index funds when assessing these sectors.”

### Overconfidence.

Next, to assess overconfidence, participants estimated their ability to outperform the market over the following 12 mo in percentage points using the same measure as in Study 1.

### Trading Frequency.

Participants then completed an incentive-compatible trading frequency measure. They responded to the following prompt using a slider from 1 to 100 trades:

“In the full study, you will trade individual stocks. Each trade will cost $1. Please indicate how many trades you would like to make each month if you are selected. For instance, if you choose 10, then you will be able to make 10 trades per month and will have $10 deducted from your account each month. Please choose carefully, as you will not be able to purchase more trades, and you will not receive a credit for these trades if you do not use all of them.”

Participants that were selected into the “larger study” were endowed with $500 minus the number of trades per month. For instance, one participant indicated four trades per month and was endowed with $488 (i.e., $500 minus 3 mo × 4 trades). These participants then traded this amount on their chosen platform and reported their results back to us in the form of anonymized financial statements. The overconfidence measure and trading frequency measure were on the same page in an order that was randomized for each participant.

### Manipulation Check.

To check the success of the manipulation, participants recalled their total stock returns from 2018 from memory in a text box that ranged from −100 to 100%.^††^ Participants were specifically asked to not look at their financial document when recalling this return. We expected that if the manipulation was successful, participants in the treatment condition should report lower returns.

Lastly, participants provided demographic variables including age, gender, income, total investment assets, portfolio composition, number of stocks owned, and whether they have a financial advisor. As a truthfulness check, we asked participants if they accessed their financial statements in the previous section. Participants were then debriefed.

## Results

### Manipulation Check.

Indicating a successful manipulation, the reported return in 2018 was lower in the treatment condition (11.0%) compared to the control condition [15.8%, *t*(364) = 2.39, *P* = 0.017, *d* = 0.25]. Participants were not significantly more likely to report accessing their financial statement in the treatment condition (86.3%) compared to the control condition [87.0%, χ^2^ (1) = 0.047, *P =* 0.828]. The following results do not substantively change if the participants that reported not accessing their financial statements are excluded from the analysis.

### Overconfidence.

Investors in this sample predicted that they would outperform the S&P 500 by 7.6% on average, significantly greater than 0% [SD = 11.0%, *t*(365) = 13.18, *P* < 0.001], indicating significant overconfidence. As predicted, overconfidence was significantly lower in the treatment condition (M = 5.8%, SD = 11.2%) compared to the control condition [M = 9.2%, SD = 10.6%, *t*(364) = 2.91, *P* = 0.004, *d* = 0.30].

### Trading Frequency.

As predicted, the intention to trade frequently was significantly lower in the treatment condition (M = 13.2 trades, SD = 12.7) compared to the control condition [M = 16.4 trades, SD = 14.2, *t*(364) = 2.26, *P* = 0.024, *d* = 0.24].

### Mediation.

We next conducted an exploratory analysis to examine whether the relationship between condition and trading frequency could be statistically explained by overconfidence. We tested this using a structural equation model with trading frequency as the dependent variable, condition as the independent variable, and overconfidence as the mediator variable. We calculated indirect pathways using bootstrapped SEs (10,000 resamples). We found a statistically reliable indirect effect through overconfidence, b = 1.75, bias-corrected 95% CI = [0.63; 3.05], *P* = 0.004. Refer to *SI Appendix* for additional details and assumptions.

## General Discussion

Investors were biased to recall their past trading performance as better than achieved. They demonstrated both a positive distortion of returns and selective forgetting. Both types of memory bias were associated with overconfidence and trading frequency. When memory bias was mitigated by having investors look up prior returns, overconfidence and trading intentions were reduced.

These findings are consistent with the interpretation that memory distortion and selective forgetting both influence overconfidence. However, we cannot fully rule out self-presentation bias as an alternative interpretation (34, 35). Based on this account, investors with a self-presentation motive would claim their past returns as higher than achieved and report a better anticipated performance. In Studies 1 and 2, the correlation between memory bias and overconfidence could be explained by self-presentation bias. This account is less able to explain Study 3, in which we manipulated memory and included an incentive-compatible measure of trading frequency. However, it still could be the case that looking up returns in Study 3 reduced overconfidence and trading frequency by deflating participants’ self-image or by reducing their ability to brag.

Though our focus was on the substantively important domain of investing, memory biases are likely to be associated with overconfidence in many domains. For instance, workers overestimate their productivity (36), chief executive officers overestimate the success of corporate investments (37), experienced chess and poker players overestimate their future performance (38), and people overestimate how quickly they can finish simple tasks (39). Overconfidence in these domains is surprising because repeated feedback offers decision-makers the opportunity to update their beliefs about the future. Biased memory may explain why they are not better calibrated. We note, however, that memory biases cannot explain all forms of overconfidence. For instance, people tend to overestimate the probability that they have answered a trivia question correctly (14) and provide too narrow of a range of possible outcomes when assessing a quantity (33). Biased memory for past performance is not likely to contribute to phenomena like these.

While a great deal of research has examined the consequences of investor overconfidence, there is little research on how to reduce it. This is surprising since overconfidence is so costly to individual investors (1) and to the financial system as a whole (40, 41). Outside of investing, several methods have proved effective at reducing overconfidence including considering an alternative option (14), taking an outside perspective (42), and considering unknowns (20). We suspect that none of these approaches would be particularly effective at reducing the confidence of an investor whose biased memory supports an inflated sense of his or her investing abilities. Debiasing memory may be more effective, and we demonstrated one way to do it. Simply making investors aware of their past returns reduces overconfidence, though it does not eliminate it completely. Brokers could easily implement this intervention by displaying prior returns on their trading platforms. Likewise, policy makers could require financial institutions to provide customers with past return information on a regular basis. Investors would be better positioned to protect and grow their wealth if they had a more accurate sense of their own trading history.

## Supplementary Material

Supplementary File

## Data Availability

Experimental data have been deposited in the Open Science Framework (10.17605/OSF.IO/N9DZF) (43).

## Footnotes

*A potential argument against measuring overplacement using this method is that it does not take portfolio risk into account, and investors who take more financial risk are correct in expecting higher returns. Merkle (2017) accounted for portfolio risk by adjusting investor’s estimates of market relative performance for the relative Sharpe ratio between investor portfolios and the stock market. This secondary measure was highly correlated with the unadjusted measure of market relative performance.

^†^Inputs could take values between −250 and 250%. We determined this to be a reasonable range to capture most investment returns based on pretesting. We included negative returns below −100% to account for the possibility that participants reported an investment in which losses exceeded the principal invested (e.g., short sales or certain options trades).

^‡^Following ref. 6, we analyze this measure of trading frequency as an interval scale.

^§^Any analysis labeled as exploratory was not preregistered. All other analyses were preregistered.

^¶^We note that in Study 1, participants could report a realized trade (i.e., a trade that was bought and sold during the study period) or an unrealized investment (i.e., a long-term investment that was purchased before the study period and/or sold after the study period), whereas in Study 2, we asked participants to only report realized trades. We made this change to ensure participants were recalling losses and gains that had occurred during the study period in Study 2.

^#^26 trades had returns of 0% and were excluded from the analysis. The following results do not meaningfully change if these trades are classified as gains or losses.

^‖^Trades were classified as gains or losses based on the return reported in the statement phase. We did this as a conservative measure to remove any effect of distortion that could have caused a trade return to be mistakenly reported as a gain in the memory phase.

**We also preregistered an analysis in which the individual-level selective forgetting dependent variable would be calculated based on the log odd coefficient for each participant from a logistic regression with whether the trade was forgotten (0: remembered, 1: forgotten) as the dependent variable and whether the trade was actually a gain or a loss (0: loss, 1: gain) as the independent variable. For 44 of the participants, we were unable to calculate this variable in a logistic regression due to insufficient variation in the independent variable. Thus, we relied on the described analysis plan, which was also preregistered as a secondary analysis.

^††^We reduced the range from prior studies in which we measured individual investment since the return of a total portfolio ought to be less extreme than individual investments because of the effect of diversification.
