# Supplementary material for: Investor memory of past performance is positively biased and predicts overconfidence
Source: Proc Natl Acad Sci U S A. 2021 Sep 2;118(36):e2026680118. doi: 10.1073/pnas.2026680118 (PMC8433511; doi:10.1073/pnas.2026680118)
Supplement: Supplementary File [file pnas.2026680118.sapp.pdf]

PNAS
www.pnas.org

Supplementary Information for

Investor Memory of Past Performance is Positively Biased and Predicts Overconfidence

Daniel J. Walters*

Philip M. Fernbach

Daniel J. Walters (daniel.walters@insead.edu), assistant professor, INSEAD Singapore, 1 Ayer Rajah Ave, Singapore 138676

Philip M. Fernbach (Philip.fernbach@colorado.edu), associate professor, University of Colorado at Boulder, Leeds School of Business, 419 UCB, Boulder CO 80309

* Corresponding author

Email: daniel.walters@insead.edu

This PDF file includes:

A. Study 1 Pre-registration 2
B. Study 2 Pre-registration 3
C. Study 3 Pre-registration 5
D. Trading Frequency Measure 6
E. Study 1 robustness check and exploratory analysis 7
F. Structural equation model paths and assumptions for Studies 1, 2, and 3 8
G. Study 3 Receipts for Investor Payments 9
H. Institutional Review Board Approval 11

A. Study 1 Pre-registration

ASPREDICTED

Wharton CREDIBILITY LAB UNIVERSITY of PENNSYLVANIA

CONFIDENTIAL - FOR PEER-REVIEW ONLY

IO Study 1 (#31934)

Created: 11/28/2019 06:47 AM (PT)
Shared: 01/20/2020 02:54 AM (PT)

This pre-registration is not yet public. This anonymized copy (without author names) was created by the author(s) to use during peer-review. A non-anonymized version (containing author names) will become publicly available only if an author makes it public. Until that happens the contents of this pre-registration are confidential.

1) Have any data been collected for this study already?
No, no data have been collected for this study yet.

2) What's the main question being asked or hypothesis being tested in this study?
Investors will have a positivity bias to recall investment returns as being higher than achieved. This tendency will be associated with overconfidence about future returns and higher trading frequency.

3) Describe the key dependent variable(s) specifying how they will be measured.
Investor overconfidence (Merkle 2013): The difference between the expected percentage return the investor expects to achieve over the next 12 months of trading and the return provided by the market.

Trading frequency (Graham et al., 2009):
How often do you trade in the financial markets? (1- At least once a day, 6 – Less than once a year)

4) How many and which conditions will participants be assigned to?
Positivity bias will be measured in a within-participant design where participants first recall what two stock investments have had the biggest monetary impact on their investment portfolios in 2019 (i.e., since January 1st 2019). They will do this task from memory and list the stock names and returns.

At the end of the survey participants will be asked to open their financial statements and answer the question again after examining their actual stock returns.

Positivity bias will be calculated as the average return reported from memory minus the average return reported from the statement.

5) Specify exactly which analyses you will conduct to examine the main question/hypothesis.
Positivity bias will be tested in a within-participant ttest comparing the average return from memory to the actual average return.

Three OLS regressions will be used to test the primary predictions. In the first regression overconfidence will be the dependent variable and positivity bias will be the independent variable. In the second regression trading frequency will be the dependent variable and positivity bias will be the independent variable. As a robustness check, both regression will also be run with additional demographic control variables including age, total investments, number of stocks owned, income, and education.

6) Describe exactly how outliers will be defined and handled, and your precise rule(s) for excluding observations.
none

7) How many observations will be collected or what will determine sample size? No need to justify decision, but be precise about exactly how the number will be determined.
Target: 400 participants

8) Anything else you would like to pre-register? (e.g., secondary analyses, variables collected for exploratory purposes, unusual analyses planned?)
no

B. Study 2 Pre-registration

ASPREDICTED

Wharton CREDIBILITY LAB UNIVERSITY of PENNSYLVANIA

CONFIDENTIAL - FOR PEER-REVIEW ONLY

IO Study 2 (#51866)

Created: 11/10/2020 05:30 PM (PT)
Shared: 12/10/2020 07:16 PM (PT)

This pre-registration is not yet public. This anonymized copy (without author names) was created by the author(s) to use during peer-review.
A non-anonymized version (containing author names) will become publicly available only if an author makes it public. Until that happens the contents of this pre-registration are confidential.

1) Have any data been collected for this study already?

No, no data have been collected for this study yet.

2) What's the main question being asked or hypothesis being tested in this study?

We predict that investors will recall past investment returns as higher than actually achieved. We predict that investors with a positivity bias in recalling returns will be more overconfident about future performance and more frequently trade stocks.

3) Describe the key dependent variable(s) specifying how they will be measured.

Positivity bias: We will measure positivity bias as the positive difference between the recalled return of a stock and the actual return of a stock. Participants will be asked to first recall the return of 10 stocks, then to open up their financial statements and report the actual return of 10 stocks.

Investor overconfidence (Merkle 2013): The difference between the expected percentage return the investor expects to achieve over the next 12 months of trading and the return provided by the market.

Trading frequency (Graham et al., 2009):
How often do you trade in the financial markets? (1- At least once a day, 6 – Less than once a year)

4) How many and which conditions will participants be assigned to?

Positivity bias will be measured in a within-participant design where participants first recall past stock returns from memory (memory condition).

At the end of the survey participants will be asked to open their financial statements and report their actual stock returns (statement condition).

5) Specify exactly which analyses you will conduct to examine the main question/hypothesis.

To test overall positivity bias we will run a repeated measure regression with stock return as the dependent variable, and memory vs. statement condition (i.e., whether the return was reported from memory or statement) as the independent variable while clustering standard errors at the participant level. Stock investments will be matched based on their rank-order. We will run a second regression and include investment amount and investment date as control variables.

To assess if distortion is the source of memory bias we will run a repeated measure regression with stock return as the dependent variable, and memory vs. statement condition (i.e., whether the return was reported from memory or statement) as the independent variable while clustering standard errors at the participant level. Stock investments will be matched based on participant's indication that the listed investment is the same in the memory and statement task (i.e., we will only evaluate matching investments that are reported in both the memory and statement recall tasks). We will run a second regression and include investment amount and investment date as control variables. To get an individual measure of distortion we will extract the participant level beta in this regression.

To assess if selective forgetting is the source of bias we will examine a repeated measure logistic regression with whether or not a trade reported from the financial statements was remembered as a binary DV. The independent variable will be whether the return is a gain or a loss while clustering standard errors at the participant level. We will run a second regression and include investment amount, investment date, and investment magnitudes (ie, absolute percentage return) as control variables. To get an individual measure of selective forgetting we will extract the participant level beta in this regression.

We will then run two sets of regressions. In the first regression overconfidence will be the dependent variable and individual level distortion and individual level selective forgetting will be the independent variables. In the second regression trading frequency will be the dependent variable and individual level distortion and individual level selective forgetting will be the independent variables. will be the dependent variable and positivity bias will be the independent variable. As a robustness check, both regression will also be run with additional demographic control variables including age, total investments, number of stocks owned, income, and education.

6) Describe exactly how outliers will be defined and handled, and your precise rule(s) for excluding observations.

We will winzorize stock returns to 500% for returns greater than 500% and -500% for returns less than -500%.

7) How many observations will be collected or what will determine sample size? No need to justify decision, but be precise about exactly how the number will be determined.
We will set a sample size target of 150 participants.

8) Anything else you would like to pre-register? (e.g., secondary analyses, variables collected for exploratory purposes, unusual analyses planned?)
As a robustness check we will calculate a mean bias by subtracting the returns from memory from the returns from statement for each of the participant's investments. We will take an average for each participant and test if this bias is greater than 0 in a ttest.

As a robustness check we will calculate a mean bias from distortion by subtracting the returns from memory from the returns from statement for each of the participant's investments recalled in both tasks. We will take an average for each participant and test if this bias is greater than 0 in a ttest.

As a robustness check we will test selective forgetting score by taking the difference between the number of losses (i.e., negative returns) and gains (i.e., positive returns) reported in the returns from memory. We will compare this difference to the same difference for stocks reported in returns from statements. If the former number is reliably larger than the latter, this will be evidence of selective forgetting for investment losses.

C. Study 3 Pre-registration

ASPREDICTED

Wharton CREDIBILITY LAB
UNIVERSITY of PENNSYLVANIA

CONFIDENTIAL - FOR PEER-REVIEW ONLY

IO Study 3 (#31930)

Created: 11/28/2019 06:01 AM (PT)
Shared: 01/21/2020 03:50 AM (PT)

This pre-registration is not yet public. This anonymized copy (without author names) was created by the author(s) to use during peer-review. A non-anonymized version (containing author names) will become publicly available only if an author makes it public. Until that happens the contents of this pre-registration are confidential.

1) Have any data been collected for this study already?
No, no data have been collected for this study yet.

2) What's the main question being asked or hypothesis being tested in this study?
When investors are made aware of their actual investment returns over a prior period they will become less overconfident about future performance and trade less frequently.

3) Describe the key dependent variable(s) specifying how they will be measured.
Investor overconfidence (Merkle 2013): The difference between the expected percentage return the investor expects to achieve over a 3 month trading period and the return provided by the market over this same period.

Trading frequency: This study will be a pretest for a larger investment study where some participants will be given a real portfolio to manage. In this study participants will pre-commit to purchasing a specific number of stock trades per month. The number of trades purchased will be the measure of intended trading frequency.

4) How many and which conditions will participants be assigned to?
Two between-participant conditions.

In the treatment participants will be asked to open their financial statement and report the actual returns of the two investments that had the biggest monetary impact on their portfolio in 2018. This is designed to make participants aware of their actual performance and debais any positivity bias in the recall of past performance.

In the control condition participants will be asked to open their financial statement and report the two sectors where they held the largest percentage of their stock investments in 2018. This condition is designed to require a similar level of effort and expose participants to similar information as the treatment condition without making participants aware of past performance.

5) Specify exactly which analyses you will conduct to examine the main question/hypothesis.
The two primary DVs will be analyzed in unpaired ttests.

6) Describe exactly how outliers will be defined and handled, and your precise rule(s) for excluding observations.
none

7) How many observations will be collected or what will determine sample size? No need to justify decision, but be precise about exactly how the number will be determined.
Target: 400 participants or 3 weeks of data collection

8) Anything else you would like to pre-register? (e.g., secondary analyses, variables collected for exploratory purposes, unusual analyses planned?)
no

D. Trading Frequency Measure

Study 1:

How often do you trade in the financial markets?

( ) At least once a day

( ) At least once a week

( ) At least once a month

( ) At least once a quarter

( ) At least once a year

( ) Less than once a year

Study 2:

How often do you trade in the financial markets?

Select the options that best describes you below.

( ) Five times a day or more

( ) Twice a day

( ) Once a day

( ) Twice a week

( ) Once a week

( ) Twice a month

( ) Once a month

( ) Once a quarter

( ) Once a year

( ) Less than once a year

E. Study 1 robustness check and exploratory analysis

Memory bias robustness check

We also conducted a robustness check to test for bias in Study 1. The bias held after performing an inverse hyperbolic sine transformation of stock returns for stock 1 ($t(410) = 3.18$, $p = .002$, $d = .22$) and stock 2 ($t(410) = 4.07$, $p < .001$, $d = .28$). Across both stocks, participants recalled a higher return than objectively achieved (40.5% of cases) more frequently than a lower return (25.8% of cases, binomial test, $p < .001$), and more frequently than the actual value (33.7% of cases, binomial test, $p = .026$).

Origin of bias exploratory analysis

We also performed an exploratory analysis to examined whether the bias in Study 1 occurred as a result of selective forgetting (i.e., forgetting about a stock with a lower return), or distortion (i.e., falsely remembering that a specific stock had a higher return). We identified selective forgetting when a participant indicated that the stock recalled in part 1 was replaced with a new stock with a lower return in part 2, and distortion when a participant indicated that the stock recalled in part 1 was the same as part 2, but that the return was lower. In cases where a participant replaced the stock (n = 235, 29.0% of cases), the recalled stock had a higher return than the forgotten stock (49.4% of cases) more frequently than a lower return (34.0% of cases, binomial test, $p = .012$), or the same return (13.6% of cases, binomial test, $p < .001$). In cases where a participant did not replace the stock (n = 576, 71.0% of cases), the return was falsely remembered as higher (36.8% of cases—a positive distortion) more frequently than lower (22.2% of cases—a negative negative, binomial test, $p < .001$), and at a similar rate to an accurate memory (41.0% of cases, binomial test, $p = .277$). We then examined if the size of the bias was larger when participants replaced the stock (i.e., when selective forgetting would drive a positivity bias) compared to when the same stock was maintained (i.e., when distortion would drive a positivity bias) in a robust regression with bias (calculated as first return minus second return) as the dependent variable and bias type as the independent variable (1 = distortion, 2 = selective forgetting) while clustering standard errors by participant. Positivity biases were marginally larger in cases of selective forgetting than distortion, $b = 8.2$ 95% CI = [-0.02; 16.4], $p = .051$. However, the positivity bias was significantly greater than 0 both in cases of false memory ($M_{bias} = 3.5\%$, SD = 34.9%, clustered $t = 2.00$, $p = .046$, $d = .14$) and cases of distortion ($M_{bias} = 11.7\%$, SD = 53.0%, clustered $t = 3.04$, $p = .003$, $d = .31$). In sum, this suggests that selective forgetting and distortion both contribute to a positivity bias in the recall of stock returns.

F. Structural equation model paths and assumptions for Studies 1, 2, and 3

STUDY 1

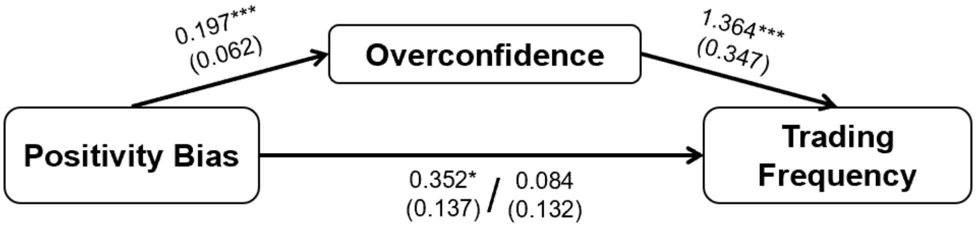

Indirect effect of Positivity Bias, b = 0.268, 95% CI = [0.090, .503], p = .011

STUDY 2

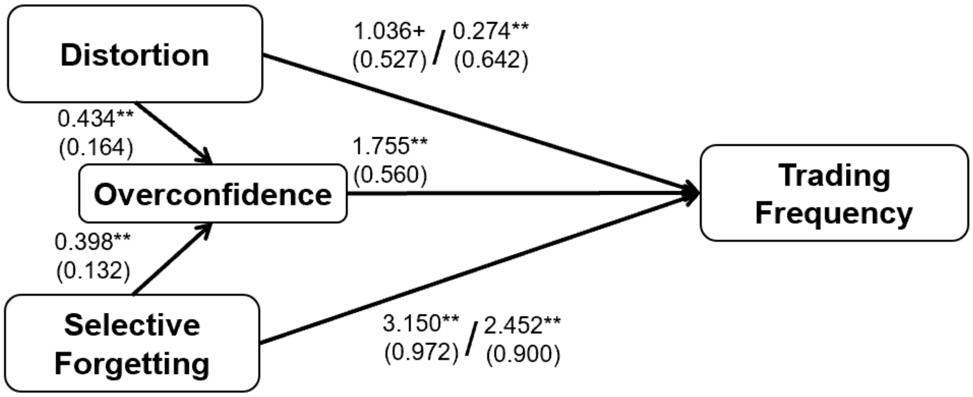

Indirect effect of distortion, b = 0.76, 95% CI = [-0.02; 1.66], p = .085; selective forgetting, b = 0.70, 95% CI = [0.12; 1.45], p = .042

STUDY 3

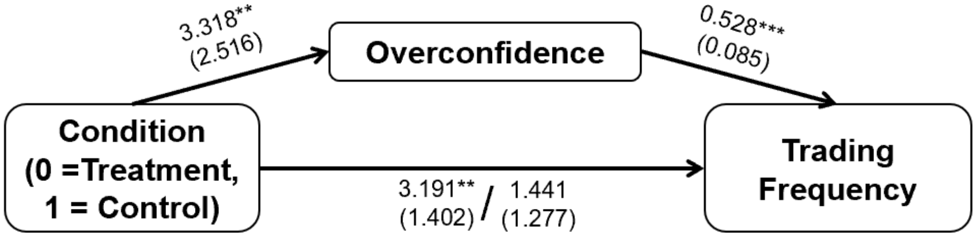

Indirect effect of Condition, b = 1.75, 95% CI = [0.63; 3.05], p = .004

Notes: Structural equation model calculated using 10,000 bootstraps in Stata v15 SEM command. Bootstrapping model assumes data is randomly sampled and independent. Bias corrected confidence intervals are presented.
+ p < .01, * p < .05, ** p < .01, *** p < .001

G. Study 3 Receipts for Investor Payments

First investor payment with identifying information blacked out:

[illegible]

Paid with

VISA Debit Card x-4382 $677.96

You'll see "PAYPAL *[redacted]" on your card statement.

Exchange rate

$677.96 SGD = $488.00 USD

1 SGD = 0.7198 USD

Ship to

[redacted]

Singapore 238255

Singapore

Transaction ID

8HV27349AR806633X

Print details

Contact info

[redacted]

Note

Funds for INSEAD Investor Study

Details

Sent to [redacted]	$488.00 USD
**Total**	**$488.00 US[illegible]**

The second participant we randomly selected elected to have his investor payment donated to the Sierra Club:

Dear [redacted]

Thank you for your gift of $440 to the Sierra Club Foundation in honor of [redacted]

Donated funds for participation in investment study

This tax deductible gift will be used to support the charitable purposes of the Sierra Club Foundation.

Your support advances climate and clean energy solutions, protects public lands and wildlife, and helps build a vibrant grassroots movement rooted in equity, inclusion, and justice.

To learn more about our work, please visit www.sierraclubfoundation.org.

Thank you again for your generous support.

Sincerely,

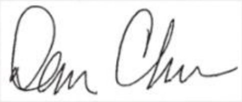

Dan Chu
Executive Director

The Sierra Club Foundation is tax exempt under Section 501(c)(3) of the Internal Revenue Code, and gifts to the Foundation are tax deductible under the Section 170(b)(1)(A)(vi) of the Code. IRS regulations require that you retain a written acknowledgement from the Foundation to support your claim of a tax deduction. The Foundation confirms that you received no goods of services in return for your gift. The Sierra Club Foundation is required by law to retain control and discretion (variance power) over all charitable funds received, including how they are disbursed within the purposes for which they were contributed; the Foundation reserves the right to provide charitable funds to recipients or beneficiaries it believes will best accomplish those purposes.

2101 Webster Street, Suite 1250 Oakland, California 94612

H. Institutional Review Board Approval

INSEAD The Business School for the World®

INSEAD UNIVERSITY
France Campus: Fontainebleau Cedex, 77305
Singapore Campus: 1 Ayer Rajah Avenue, 138676

Craig Smith, Ph. D.
CHAIR, PANEL ON HUMAN PARTICIPANTS RESEARCH

+33 (0)1 60 72 00 00

NOTICE ON FULL ETHICAL REVIEW

Date: February 17, 2021

To: Daniel WALTERS, Faculty, INSEAD

From: Craig Smith, PhD, Chair, Institutional Review Board on Human Participants Research

Protocol: Judgments and Choices Under Uncertainty

Protocol ID: 2017-14Nov-WALTERS

The INSEAD ethical review committee has reviewed your research protocol submitted on 14 November 2017 and the amendment submitted on 22 December 2017 and determined that the study is **approved** with the request that the voluntariness of participation be stressed when it is announced in class to comply with consent requirements.
If this protocol is used in conjunction with any other human use, it must be re-reviewed. The ethical committee requests prompt notification of any complications or incidents of noncompliance, which may occur during any human use procedure.

Please remember that in case any new data is collected, all data including the consent forms must be retained for a minimum of three (3) years past the completion of this research. Additional requirement may be imposed by your funding agency, your department, or other entities.

Yours sincerely,

Craig Smith

Craig Smith

Review type: FULL – NEW

Europe Campus – Boulevard de Constance
77305 Fontainebleau Cedex, France
Tel: +33 (0)1 60 72 40 00 Fax: +33 (0)1 60 74 55 00/01

www.insead.edu

Institut privé d'enseignement supérieur Association loi 1901
APE 8542Z SIRET 775 703 390 000 10 TVA FR60 775 703 390
